# Co-circulation of multiple enterovirus D68 subclades, including a novel B3 cluster, across Europe in a season of expected low prevalence, 2019/20

**DOI:** 10.2807/1560-7917.ES.2020.25.2.1900749

**Published:** 2020-01-16

**Authors:** Sofie Elisabeth Midgley, Kimberley Benschop, Robert Dyrdak, Audrey Mirand, Jean-Luc Bailly, Sibylle Bierbaum, Stefan Buderus, Sindy Böttcher, Anna-Maria Eis-Hübinger, Mario Hönemann, Veronika Vorobieva Jensen, Ulla Birgitte Hartling, Cécile Henquell, Marcus Panning, Marianne Kragh Thomsen, Emma B Hodcroft, Adam Meijer

**Affiliations:** 1 Department for Virus and Microbiological Special Diagnostics, Statens Serum Institut, Copenhagen, Denmark; 2 Centre for Infectious Disease Research, Diagnostics and Laboratory Surveillance, National Institute for Public Health and the Environment (RIVM), Bilthoven, the Netherlands; 3 Department of Clinical Microbiology, Karolinska University Hospital, Stockholm, Sweden; 4 Department of Microbiology, Tumor and Cell Biology, Karolinska Institutet, Stockholm, Sweden; 5 CHU Clermont-Ferrand, Centre National de Référence des entérovirus et parechovirus - Laboratoire Associé, Laboratoire de Virologie, Clermont-Ferrand, France; 6 Université Clermont Auvergne, CNRS, Laboratoire Microorganismes: Génome et Environnement, Clermont-Ferrand, France; 7 Institute of Virology, University of Freiburg, Freiburg, Germany; 8 Department of General Pediatrics, St.-Marien-Hospital, Bonn, Germany; 9 National Reference Center for Poliomyelitis and Enteroviruses, Robert Koch-Institute, Berlin, Germany; 10 Institute of Virology, University of Bonn Medical Center, Bonn, Germany; 11 Institute of Virology, University of Leipzig, Leipzig, Germany; 12 Department of Paediatrics, Aarhus University Hospital, Aarhus, Denmark; 13 Department of Clinical Microbiology, Aarhus University Hospital, Aarhus, Denmark; 14 Biozentrum, University of Basel, Basel, Switzerland; 15 Swiss Institute of Bioinformatics, Basel, Switzerland

**Keywords:** enterovirus D68, acute flaccid myelitis, severe respiratory infection, novel strains, surveillance

## Abstract

Enterovirus D68 (EV-D68) was detected in 93 patients from five European countries between 1 January 2019 and 15 January 2020, a season with expected low circulation. Patients were primarily children (n = 67, median age: 4 years), 59 patients required hospitalisation and five had severe neurologic manifestations. Phylogenetic analysis revealed two clusters in the B3 subclade and subclade A2/D. This circulation of EV-D68 associated with neurological manifestations stresses the importance of surveillance and diagnostics beyond expected peak years.

Enterovirus D68 (EV-D68) is primarily a respiratory virus. Previously published data have suggested circulation with a biennial epidemic cycle in Europe and North America [1-3], but surveillance is not consistent. The Danish enterovirus surveillance detected two cases of EV-D68 infection in August 2019 and a further case in early September. Colleagues in other European countries were contacted, and France, Germany, the Netherlands and Sweden responded that they had also seen cases. Here we report on the start of seasonal circulation of EV-D68 in five European countries, a circulation which is still ongoing with further cases detected since the initial submission of this report.

## Epidemiological trend and description of cases

**Figure 1:**
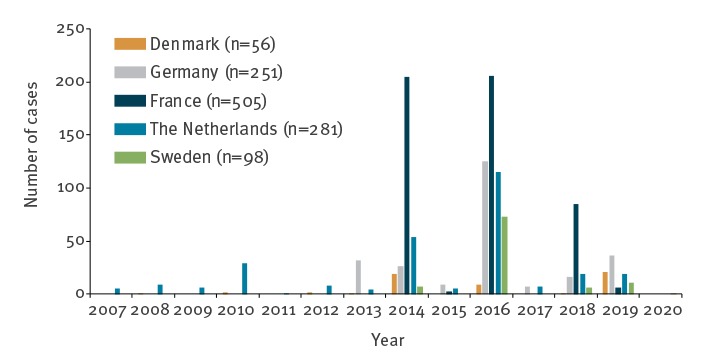
Yearly variation in detection of enterovirus D68 in five European countries, 1 January 2007–15 January 2020 (n = 1,191)

Denmark, France, Germany, the Netherlands and Sweden have seen continuous circulation of EV-D68 with variable upsurges since 2007 (Figure 1). However, in 2018, only one case was detected in Denmark, 16 in Germany, 19 in the Netherlands and six in Sweden, whereas other European countries experienced large outbreaks with between 21 and at least 114 cases (as reported by November 2018 by Public Health Wales), overall including at least 11 cases with AFM [4-8].

For Sweden, cases in 2014 include only those confirmed by sequencing, and only a limited number of samples were screened (n = 30). Data from Germany represent five sites for the period 2013 to 2018 (Bonn, Düsseldorf, Freiburg, Leipzig and Würzburg), but only three for 2019 and 2020 (Bonn, Freiburg and Leipzig).

A total of 93 EV-D68 infections were reported between 1 January 2019 and 15 January 2020 (Denmark n = 21, France n = 6, Germany n = 36, the Netherlands n = 19, Sweden n = 11). Epidemiological and clinical information was collected for cases where possible (Table 1). As surveillance samples and data are collected retrospectively, data for December 2019 and January 2020 are not complete.

**Table 1:** Cases of enterovirus D68 in five European countries, 1 January 2019–15 January 2020 (n = 93)

Case number	Sample date (month-year)	Age (years)	Sex	Hospitalised	Pre-existing disease	Travel abroad < 2 weeks before sampling	Fever	Enteric symptoms	Respiratory symptoms	Neurological symptoms	Dermatological symptoms	Co-infections
DK-01	Aug-19	15	F	Y	N	N	Y	None	Bronchiolitis	N	N	N
DK-02	Aug-19	2	F	N	N	N	Y	None	Common cold	N	N	N
DK-03	Sep-19	3	F	Y	Y	N	Y	None	Acute bronchitis	N	N	N
DK-04	Sep-19	1	M	Y	N	N	Y	None	ILI	AFM	NA	Adenovirus, parechovirus
DK-05	Sep-19	2	M	Y	Y	N	Y	None	Obstructive bronchiolitis	N	Rash	Adenovirus
DK-06	Sep-19	29	F	Y	Y	N	Y	None	Common cold	Cranial nerve palsies, dysphagia, dysatria	N	N
DK-07	Sep-19	0	F	N	N	NA	Y	None	ILI	N	Rash	N
DK-08	Sep-19	0	F	Y	N	NA	Y	Diarrhoea, rumbling stomach	Common cold	N	N	*Bordetella pertussis*
DK-09	Oct-19	0	F	Y	Y	N	Y	None	Pneumonia	N	N	*Moraxella catarrhalis*
DK-10	Oct-19	27	M	Y	Y	N	Y	Diarrhoea	Pneumonia	Reduced strength in right leg, attenuated patellar and plantar reflexes	N	Suspected bacterial infection
DK-11	Oct-19	50	F	N	NA	NA	N	None	Common cold	N	N	N
DK-12	Oct-19	30	M	N	N	N	Y	None	ILI	N	N	N
DK-13	Oct-19	0	F	Y	N	N	N	None	Pneumonia	N	N	*Haemophilus influenzae, Moraxella catarrhalis*
DK-14	Nov-19	2	M	Y	Y	N	Y	None	Pneumonia	N	N	N
DK-15	Nov-19	2	F	Y	Y	N	Y	N	Obstructive bronchiolitis	N	N	Parechovirus, adenovirus, *Haemophilus influenzae*
DK-16	Nov-19	1	M	Y	N	NA	N	Diarrhoea	N	N	N	N
DK-17	Nov-19	16	F	Y	N	N	N	Nausea, vomiting	Y	Ataxic cerebral palsy, diplopia, hypoaesthesia	N	N
DK-18	Nov-19	2	M	Y	Y	NA	Y	None	Common cold	N	N	*Haemophilus influenzae, Pseudomonas aeruginosa*
DK-19	Nov-19	2	M	Y	Y	NA	N	N	Respiratory distress	N	N	N
DK-20	Dec-19	2	F	Y	Y	NA	NA	NA	Bilateral pneumonia	N	N	*Pneumococcu*s
DK-21	Dec-19	1	M	N	Y	NA	NA	NA	Obstructive bronchiolitis	N	NA	Rhinovirus C22 *Haemophilus influenzae*
NL-01	Jan-19	61	M	NA	NA	Y	NA	NA	NA	NA	NA	NA
NL-02	Jan-19	65	F	N	Y	N	NA	Yes	N	N	N	NA
NL-03	Apr-19	1	F	Y	NA	Y	NA	NA	Respiratory insufficiency	NA	NA	NA
NL-04	Aug-19	5	F	Y	NA	Y	NA	NA	Respiratory insufficiency	NA	NA	NA
NL-05	Aug-19	75	F	NA	NA	NA	NA	NA	Pneumonia	NA	NA	NA
NL-06	Sep-19	46	M	N	N	N	Y	Diarrhoea	ILI	NA	NA	NA
NL-07	Oct-19	61	F	N	N	N	N	N	ARI, dyspnoea	NA	NA	NA
NL-08	Nov-19	1	F	NA	NA	NA	NA	N	Common cold, dyspnoea	NA	NA	NA
NL-09	Nov-19	1	M	NA	NA	N	Y	N	ARI, dyspnoea	NA	NA	Respiratory syncytial virus type A
NL-10	Nov-19	89	M	NA	NA	NA	NA	NA	NA	NA	NA	NA
NL-11	Nov-19	4	F	NA	NA	NA	NA	NA	Dyspnoea	NA	NA	NA
NL-12	Nov-19	2	F	NA	NA	N	Y	N	ARI, dyspnoea	NA	NA	NA
NL-13	Nov-19	0	M	NA	NA	NA	NA	NA	NA	NA	NA	NA
NL-14	Nov-19	2	F	NA	NA	NA	NA	NA	Pneumonia and bronchial hyperreactivity	NA	NA	NA
NL-15	Dec-19	4	M	NA	NA	NA	NA	NA	NA	NA	NA	NA
NL-16	Dec-19	53	M	NA	N	N	Y	N	ILI, dyspnoea	NA	NA	NA
NL-17	Dec-19	7	F	NA	N	N	N	N	ARI	NA	NA	NA
NL-18	Dec-19	22	M	NA	N	N	N	N	ILI	NA	NA	NA
NL-19	Mar-19	60	M	N	N	N	Y	N	ILI, dyspnoea	N	NA	Influenza virus A(H1N1)pdm09
SE-01	Aug-19	70	F	NA	NA	NA	NA	NA	NA	NA	NA	*Haemophilus influenzae*
SE-02	Sep-19	2	M	NA	NA	NA	NA	NA	NA	NA	NA	Rhinovirus
SE-03	Sep-19	0	F	N	N	N	Y	N	Y	N	NA	N
SE-04	Sep-19	5	F	NA	NA	NA	NA	NA	NA	NA	NA	N
SE-05	Oct-19	61	M	N	N	Y	Y	N	Y	N	NA	Streptococcus pneumoniae
SE-06	Oct-19	2	M	NA	NA	NA	NA	NA	NA	NA	NA	N
SE-07	Oct-19	4	F	Y	N	N	N	N	Y	N	NA	N
SE-08	Oct-19	2	M	Y	N	N	Y	N	Y	N	NA	Adenovirus Rhinovirus *Haemophilus influenzae*
SE-09	Nov-19	2	M	NA	NA	NA	NA	NA	NA	NA	NA	N
SE-10	Nov-19	5	M	NA	NA	NA	NA	NA	NA	NA	NA	NA
SE-11	Jan-20	69	M	NA	NA	NA	NA	NA	NA	NA	NA	NA
FR-01	Oct-19	0	F	Y	N	N	Y	N	Bronchiolitis	N	N	N
FR-02	Oct-19	33	M	Y	Y	N	Y	N	Pneumonia with acute respiratory distress	N	N	Streptococcus pneumoniae
FR-03	Nov-19	1	M	Y	N	N	Y	Diarrhoea	N	AFM	N	N
FR-04	Nov-19	0	F	Y	N	N	Y	Diarrhoea	Bronchiolitis	N	N	N
FR-05	Dec-19	51	F	Y	N	Y	Y	N	Bronchitis	N	N	N
FR-06	Dec-19	66	M	Y	Y	N	Y	N	Respiratory distress	N	N	N
DE-01	Jul-19	1	M	Y	N	N	Y	N	Obstructive bronchitis	N	N	N
DE-02	Sep-19	1	M	Y	N	N	Y	N	Obstructive bronchitis	N	N	Rhinovirus C17
DE-03	Sep-19	20	F	Y	Y	N	Y	Y	Y	N	N	Multiple bacterial and viral infections
DE-04	Sep-19	9	M	Y	Y	N	Y	N	Asthma exacerbation	N	N	N
DE-05	Sep-19	1	M	Y	N	N	Y	N	Obstructive bronchitis	N	N	Rhinovirus A49
DE-06	Oct-19	0	M	Y	N	N	N	N	Obstructive bronchitis	N	N	N
DE-07	Oct-19	0	F	Y	N	N	Y	N	Y	N	N	N
DE-08	Oct-19	6	F	Y	N	N	Y	N	Y	Bilateral lower limb paralysis	N	N
DE-09	Oct-19	12	M	Y	N	NA	Y	NA	NA	NA	NA	N
DE-10	Nov-19	2	M	Y	N	N	Y	N	Obstructive bronchitis	N	N	N
DE-11	Nov-19	7	F	Y	Y	N	Y	N	Asthma exacerbation	N	N	N
DE-12	Nov-19	2	M	Y	N	N	Y	N	Obstructive bronchitis	N	N	N
DE-13	Nov-19	5	F	Y	Y	N	Y	N	Acute bronchitis	Seizures	N	*Clostridioides difficile*
DE-14	Nov-19	22	F	Y	N	N	N	N	Obstructive bronchitis	N	N	N
DE-15	Nov-19	1	F	Y	N	N	Y	N	Acute bronchitis	N	N	N
DE-16	Nov-19	8	F	Y	Y	NA	NA	N	Y	NA	NA	Adenovirus
DE-17	Nov-19	78	F	N	Y	N	N	N	Y	N	N	N
DE-18	Nov-19	8	M	Y	N	NA	NA	N	Y	NA	N	N
DE-19	Nov-19	0	F	Y	Y	N	Y	N	Y	N	N	N
DE-20	Nov-19	5	F	Y	N	N	N	N	Obstructive bronchitis	N	N	N
DE-21	Nov-19	4	F	Y	Y	N	Y	N	Y	N	N	N
DE-22	Nov-19	2	M	Y	NA	NA	Y	NA	Y	NA	NA	N
DE-23	Nov-19	29	M	Y	Y	N	N	N	Y	N	N	N
DE-24	Nov-19	7	M	Y	NA	NA	NA	NA	Y	NA	NA	N
DE-25	Oct-19	74	F	Y	Y	N	N	N	Y	N	NA	N
DE-26	Nov-19	71	M	N	Y	Y	N	N	N	N	N	N
DE-27	Nov-19	1	M	Y	N	N	N	N	Obstructive bronchitis	N	N	N
DE-28	Nov-19	3	M	Y	N	NA	Y	N	Y	N	N	N
DE-29	Nov-19	40	F	N	N	NA	Y	N	Y	NA	NA	N
DE-30	Dec-19	2	F	Y	N	N	N	N	Obstructive bronchitis	N	N	N
DE-31	Dec-19	1	F	Y	N	N	Y	N	Obstructive bronchitis	N	N	N
DE-32	Dec-19	6	M	Y	N	N	Y	N	Pneumonia	N	N	N
DE-33	Dec-19	2	M	Y	N	N	Y	N	Bronchitis and pneumonia	N	N	Rhinovirus C55
DE-34	Dec-19	1	M	Y	N	N	N	Y	Pneumonia	N	N	Bocavirus 1 Respiratory syncytial virus
DE-35	Dec-19	5	F	Y	Y	N	N	N	Obstructive bronchitis	Seizures	N	N
DE-36	Dec-19	2	M	Y	N	Y	N	N	URTI	N	N	N

AFM: acute flaccid myelitis; ARI: acute respiratory infection; DK: Denmark; FR: France; DE: Germany; ILI: influenza-like illness; N: no; NA: not available; NL: the Netherlands; SE: Sweden; URTI: upper respiratory infection; Y: yes.

**Figure 2:**
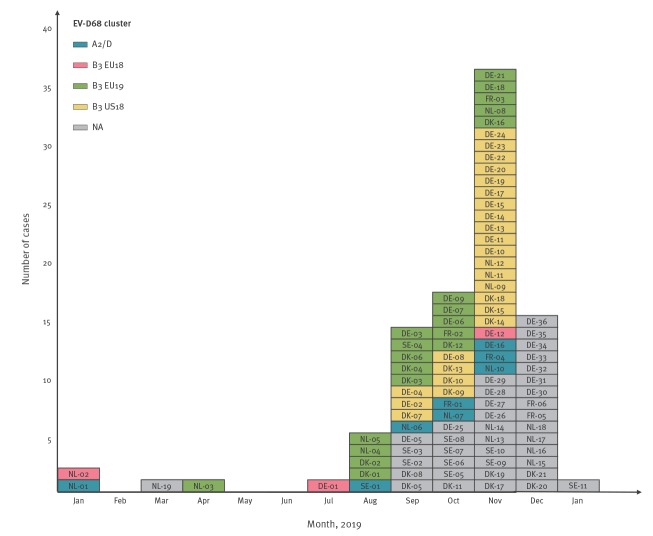
Epicurve of enterovirus D68 cases by phylogenetic cluster, five European countries, 1 January 2019–15 January 2020 (n = 93)

DK: Denmark; EU18: 2019 sequences with most recent common ancestor in large B3 cluster of European sequences from 2018; EU19: novel B3 cluster identified in this study; FR: France; DE: Germany; NA: sequence data for clade allocation not available or phylogenetic analysis not done; NL: the Netherlands; SE: Sweden; US18: 2019 sequences with most recent common ancestor in large B3 cluster predominantly containing sequences from the United States in 2018.

Cases were identified through enterovirus surveillance (n = 31), rhinovirus surveillance (n = 3), influenza and respiratory infection community surveillance (n = 12) or hospital-based diagnostics of respiratory infections (n = 47). Following diagnostic testing of respiratory samples for enterovirus and/or rhinovirus using commercial or in-house assays, EV-D68 cases were identified by either EV-D68-specific real-time PCR or partial sequencing of the VP1 and/or VP4/VP2 region of the genome [9-11]. Most of the cases were identified during the usual enterovirus season starting in late summer (August n = 5, September n = 14, October n = 17, November n = 36 and December n=15; Figure 2).

For clade allocation of cases see section *Phylogenetic analysis of enterovirus D68 strains*.

Of the Dutch cases, two became ill in Turkey and were diagnosed with EV-D68 after being hospitalised for respiratory support upon their return to the Netherlands. One Swedish patient had a recent travel history to East Asia and one French patient had travelled to Portugal. Most of the cases were children (n = 67), with a median age of 4 years (range: 16 days–89 years). Forty-six patients were female.

## Clinical manifestations of cases

Five patients, from Denmark, France and Germany, presented with severe neurological symptoms. Of these, one was a 1 year-old, previously healthy boy who presented with acute flaccid myelitis (AFM) following a febrile respiratory infection. The paralysis was asymmetric, included all four limbs and the torso, with severe paraesthesia in affected limbs. The second patient was a 15-month-old boy who presented with AFM following a digestive prodromal illness. The third patient was a 29-year-old woman who presented with cranial nerve palsy. She underwent a caesarean section in week 40 of pregnancy because of the acute neurological symptoms. The fourth patient was a 6-year-old girl who presented with paralysis of both legs and the bladder. The fifth patient was a 16-year-old girl who presented with loss of balance and coordination, double vision and loss of sensation. In addition to the severe neurological cases, one further patient, a 27-year-old man, presented with a discrete unilateral paresis of the right leg. Two patients suffered seizures, most likely related to underlying conditions.

The clinical manifestations for the remaining patients ranged from mild cold-like symptoms of the upper respiratory tract to severe pneumonia requiring continuous positive airway pressure and respirator support (Table 1). Fifty-nine patients (among the 73 for whom this information was available) required hospitalisation, either because of severity of symptoms or underlying medical conditions. Twenty-one patients had underlying medical conditions, including asthma, cancer, non-HIV-related immunodeficiency, epilepsy and trisomy 14 mosaicism with growth retardation, cognitive impairment and multiple malformations. For one Dutch case, EV-D68 was identified in bronchoalveolar lavage and this patient also had a pneumococci-positive antigen test in urine. The patient died after 11 days of hospitalisation from bilateral pneumonia.

## Phylogenetic analysis of enterovirus D68 strains

Full- or nearly full-length genome sequencing was successfully carried out for seven strains (Denmark and the Netherlands: in-house protocols, Sweden [12]). Sequences which were available up to and including 6 December 2019 are available on GenBank (Table 2).

**Table 2:** GenBank accession numbers and available sequence for phylogenetic analysis, enterovirus D68 strains from five European countries, 1 January–6 December 2019 (n = 67)

Case number	GenBank accession number	Sequence
DK-01	MN896974	Partial genome
DK-02	MN896975	Partial genome
DK-03	NA	NA
DK-04	MN896976	Partial VP1
DK-05	NA	NA
DK-06	MN896977	Partial VP1
DK-07	MN896978	Full genome
DK-08	NA	NA
DK-09	MN896979	Partial VP4/VP2
DK-10	MN896980	Partial VP1
DK-11	NA	NA
DK-12	MN896981	Partial VP1
DK-13	MN896982	Partial VP1
DK-14	MN896983	Partial VP1
DK-15	MN896985	Partial VP1
DK-16	MN896984	Partial VP1
DK-17	NA	NA
DK-18	MN896986	Partial VP1
NL-01	MN764886	Partial VP1
NL-02	MN764887	Partial VP1
NL-03	MN764888	Partial VP1
NL-04	MN764889	Partial VP1
NL-05	MN726800	Full genome
NL-06	MN726801	Full genome
NL-07	MN726798	Full genome
NL-08	MN726799	Partial VP1
NL-09	MN809623	Complete VP1
NL-10	MN809624	Partial VP1
NL-11	MN809625	Partial VP1
NL-12	MN809626	Partial VP1
SE-01	MN935869	Full genome
SE-02	NA	NA
SE-03	NA	NA
SE-04	MN935870	Full genome
SE-05	NA	NA
SE-06	NA	NA
SE-07	NA	NA
SE-08	NA	NA
SE-09	NA	NA
FR-01	LR743438	Complete VP1
FR-02	LR743439	Complete VP1
FR-03	LR743440	Complete VP1
FR-04	LR743441	Complete VP1
DE-01	MN814240	Partial VP1
DE-02	MN814241	Partial VP1
DE-03	MN814242	Partial VP1
DE-04	MN814243	Partial VP1
DE-05	NA	NA
DE-06	MN814244	Partial VP1
DE-07	MN832475	Partial VP4/VP2
DE-08	MN812202	Partial VP1
DE-09	MN832476	Partial VP4/VP2
DE-10	MN814245	Partial VP1
DE-11	MN814246	Partial VP1
DE-12	MN814247	Partial VP1
DE-13	MN814248	Partial VP1
DE-14	MN814249	Partial VP1
DE-15	MN814250	Partial VP1
DE-16	MN832477	Partial VP4/VP2
DE-17	MN832478	Partial VP4/VP2
DE-18	MN832479	Partial VP4/VP2
DE-19	MN832480	Partial VP4/VP2
DE-20	MN814251	Partial VP1
DE-21	MN832481	Partial VP4/VP2
DE-22	MN832482	Partial VP4/VP2
DE-23	MN832483	Partial VP4/VP2
DE-24	MN832484	Partial VP4/VP2

NA: not available.

**Figure 3:**
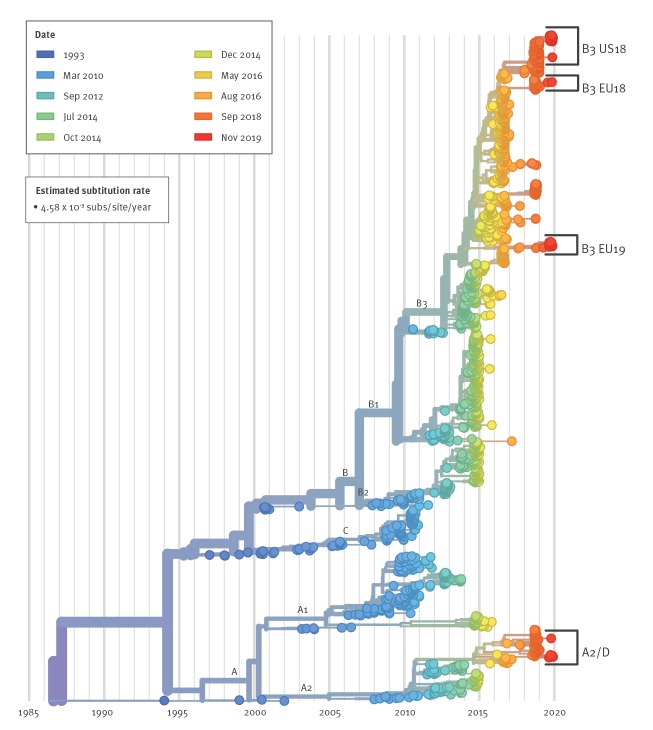
Phylogenetic analysis of enterovirus D68 with Nextstrain using partial VP1 sequences (n = 1,693)

Phylogenetic analysis of VP1 sequence data was carried out using the Nextstrain augur pipeline [13]. We included samples from this study with ≥ 300 bp in VP1 (Table 2), alongside all available VP1 sequences in GenBank of ≥ 700 bp, randomly down-sampled to 20 samples per country per month to avoid sampling bias and overrepresentation of some countries (particularly during the 2014 and 2016 epidemics; no 2019 samples were down-sampled). The code is available at https://github.com/enterovirus-phylo/evd68-2019; the analysis can be viewed at https://nextstrain.org/community/enterovirus-phylo/evd68-2019/vp1-300. This analysis will be updated with new sequence data as this becomes available. EV-D68 has been characterised into the major clades A, B, and C, with A and B divided into the subclades A1–A2, and B1–B3. Some studies designate A2 as D and subdivide it into D1 and D2 (Figure 3).

EU18: 2019 sequences with most recent common ancestor in large B3 cluster of European sequences from 2018; EU19: novel B3 cluster identified in this study; US18: 2019 sequences with most recent common ancestor in large B3 cluster predominantly containing sequences from the United States in 2018.

For sequences with a sample date up to and including 2018, VP1 fragments of ≥ 700 bp in length were included, for sequences from 2019, fragments of ≥ 300 bp were included.

Divergence between major subclade divisions is shown, sequences are colour-coded according to date of sampling. Brackets identify where sequences reported in this study cluster with previous samples.

**Figure 4:**
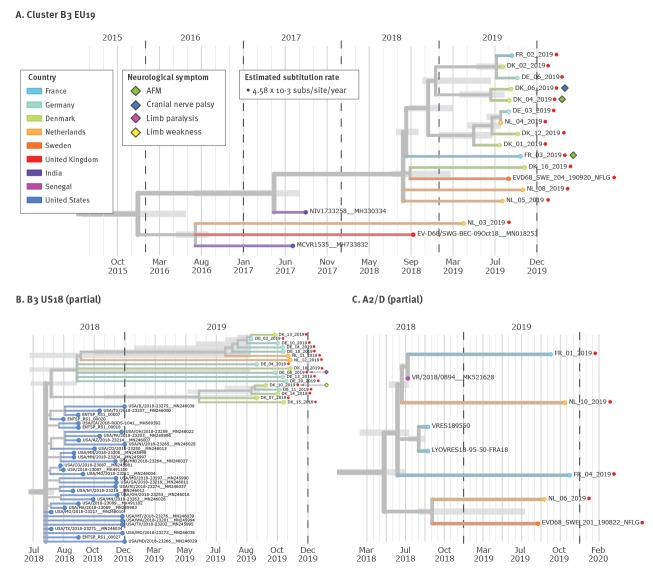
Phylogenetic analysis of three clusters of partial VP1 sequences of enterovirus D68 strains using Nextstrain, five European countries, 1 January–6 December 2019 (n = 36)

The dominant subclades have varied between years of upsurge: the 2014 outbreak was dominated by B1 strains, whereas the 2016 and 2018 outbreaks consisted only of the B3 and A2/D subclades. Some clades and subclades may no longer be circulating, as evidenced by the lack of clade C strains since 2010 and the absence of A1, B1 and B2 strains since 2014/15. In the current study, there was a close genetic relationship between 15 strains from all five countries (Figure 4A). These formed a distinct cluster within clade B3 (EU19 in Figures 2 and 3), which did not include previously detected B3 strains from these countries and was not well represented in the 2016 or 2018 epidemic (see Nextstrain analysis, https://nextstrain.org/community/enterovirus-phylo/evd68-2019/vp1-300). Fourteen of the sequences clustered closely, with an estimated most recent common ancestor (MRCA) in September 2018, suggesting that this cluster may have circulated in Europe during the 2018 season. Four German strains clustered within this group in the VP4/VP2 region (https://nextstrain.org/community/enterovirus-phylo/evd68-2019/vp4vp2). NL-03 (infection presumably acquired in Turkey) was more distant, with an estimated MRCA with the larger 2019 European B3 subclade of January 2016.

AFM: acute flaccid myelitis.

Zoomed in view of three phylogenetic clusters containing 2019 EV-D68 sequences. Nodes are coloured by country; the colour legend is the same for all three panels. Date estimate confidence intervals are shown as light grey bars for nodes leading to 2019 strains. Years are marked with dashed vertical lines. Red dots: sequences from this study; diamond shapes: neurological symptoms for 2019 cases.

Panel A shows a novel 2019 B3 cluster which is poorly represented in previous years. Panel B shows two 2019 clusters which descend from a large, United States-dominated, 2018 B3 cluster. The partial VP1 sequences of the upper seven strains with nodes having wide date estimate confidence intervals are identical. Panel C shows 2019 A2/D strains clustering with strains that circulated in France and Senegal in 2018.

Seventeen other strains (US18 in Figures 2 and 3) formed two further clusters within clade B3, one cluster with five strains and an MRCA of May 2019, the other with 12 strains and an MRCA of September 2018 (Figure 4B). These two clusters had a common MRCA in August 2018. This common ancestor fell within a large American cluster circulating in 2018, which previously contained only three non-American sequences. Strains NL-09, DK-09 and four German strains also fell within this American cluster; NL-09 separately from the other 2019 VP1 samples and the other five in the VP4/VP2 region (Figure 2, sequence data not shown). Samples DE-01 and DE-12 formed a pair and were descended from a cluster of B3 strains that circulated in Europe in 2018, including one of the Dutch cases detected in January (NL-02; cluster EU18 in Figures 2 and 3). The other Dutch case from January (NL-07; cluster A2/D in Figures 2 and 3), along with five other strains, belonged to clade A2/D (Figure 4C) and rooted, although separately, in a cluster circulating in Europe in autumn 2018. Five and 21 sequences, respectively, were long enough to be included in the Nextstrain full-length (≥ 6,000 bp) and VP1 (≥ 700 bp) builds; they can be viewed at https://nextstrain.org/enterovirus/d68/genome and https://nextstrain.org/enterovirus/d68/vp1.

## Discussion

Here we report the detection of EV-D68 infections associated with both respiratory and neurological manifestations in five European countries in the autumn of 2019. Following its first identification in 1962, only 699 cases of EV-D68 were reported in scientific literature before 2014 [14]. From 2014 onwards, the epidemiology appears to have changed, and EV-D68 has increasingly been associated with outbreaks of respiratory infections and a concurrent upsurge of AFM [15,16] with poor long-term prognosis [17,18]. We identified 15 sequences, collected from five countries, which formed a distinct cluster within subclade B3 not previously described in Europe, illustrating the rapid evolution and spread of EV-D68. The sequences most closely related to this novel B3 cluster were sampled in India in 2017 and from sewage in the United Kingdom in 2018. This would suggest either a very low circulation of strains of this cluster or a lack of sustained global surveillance for EV-D68 and/or submission of sequences in GenBank, or both. The estimated MRCA in mid-2018 suggests that these viruses were already circulating and diversifying during and after the 2018 EV-D68 epidemic, but were not sampled until August 2019. In previous European seasons of EV-D68, Denmark has only reported respiratory infections caused by EV-D68, whereas Germany, France, the Netherlands and Sweden have experienced paralytic cases [11,18,19].

The EV-D68 cases were detected through routine surveillance networks in each country, with alerts to physicians posted in Denmark and the Netherlands. The number of samples processed in each country was within the expected range for the enterovirus season, but the detection rate of EV-D68 was higher than expected in Denmark, Germany and Sweden, where the number of detections for the 2018 season had been unexpectedly low. Detection rates in the other countries were similar to those seen in previous years with low circulation. The rate of hospitalisation among cases was high, however, current surveillance systems primarily allow the detection of severe infections. In this study, 12 of 93 cases were detected through community-based surveillance and a further three cases through sequencing of rhinovirus-positive samples, highlighting the importance of including such surveillance when monitoring infections such as EV-D68. The detection of EV-D68 associated disease indicates a continuous, and most likely underestimated, global circulation of this enterovirus type that was recognised after the large outbreak in the United States in 2014 had sparked increased testing. This justifies the need for reinforced enterovirus surveillance based on a more systematic screening of respiratory samples, collected in hospitalised patients or through the surveillance of respiratory infections (such as influenza community surveillance). Our report also highlights the heterogeneity of the surveillance of EV-D68 in Europe, which may hamper both the comparison of the epidemiological pattern between countries and, more importantly, the early detection of an outbreak. Continuous surveillance will add to our understanding of how EV-D68 evolves outside of outbreak periods and whether low-prevalence years vary in geographic or demographic distribution. The detection of EV-D68 in a presumed year of low activity and the presence of patients with severe neurologic symptoms, stresses the importance of continuous and systematic surveillance and availability of diagnostics for workup of clinical cases.
